# Survival Prediction in Allogeneic Haematopoietic Stem Cell Transplant Recipients Using Pre‐ and Post‐Transplant Factors and Computational Intelligence

**DOI:** 10.1111/jcmm.70672

**Published:** 2025-08-27

**Authors:** Panagiotis G. Asteris, Danial J. Armaghani, Amir H. Gandomi, Ahmed Salih Mohammed, Zoi Bousiou, Ioannis Batsis, Nikolaos Spyridis, Georgios Karavalakis, Anna Vardi, Markos Z. Tsoukals, Leonidas Triantafyllidis, Evangelos I. Koutras, Nikos Zygouris, Georgios A. Drosopoulos, Leonidas Dritsas, Nikolaos A. Fountas, Nikolaos M. Vaxevanidis, Abidhan Bardhan, Pijush Samui, George D. Hatzigeorgiou, Jian Zhou, Konstantina V. Leontari, Paschalis Evangelidis, Nikolaos Kotsiou, Ioanna Sakellari, Eleni Gavriilaki

**Affiliations:** ^1^ Computational Mechanics Laboratory School of Pedagogical and Technological Education Athens Greece; ^2^ School of Civil and Environmental Engineering University of Technology Sydney Sydney New South Wales Australia; ^3^ Faculty of Engineering & IT University of Technology Sydney Sydney New South Wales Australia; ^4^ University Research and Innovation Center (EKIK) Óbuda University Budapest Hungary; ^5^ Engineering Department American University of Iraq Sulaimani Iraq; ^6^ Hematology Department—BMT Unit G Papanicolaou Hospital Thessaloniki Greece; ^7^ Discipline of Civil Engineering University of Central Lancashire Preston UK; ^8^ Department of Electrical and Electronic Engineering Educators School of Pedagogical and Technological Education Athens Greece; ^9^ Department of Mechanical Engineering Educators School of Pedagogical and Technological Education Athens Greece; ^10^ Civil Engineering Department National Institute of Technology Patna Patna India; ^11^ Hellenic Open University Patras Greece; ^12^ Central South University Changsha China; ^13^ National Kapodistrian University of Athens Aretaieio Hospital Athens Greece; ^14^ 2nd Propedeutic Department of Internal Medicine Aristotle University of Thessaloniki Thessaloniki Greece

**Keywords:** allogeneic haematopoietic cell transplantation, artificial intelligence, graft‐versus‐host disease, machine learning, prediction model, survival

## Abstract

Advancements in artificial intelligence (AI) predictive models have emerged as valuable tools for predicting survival outcomes in allogeneic haematopoietic stem cell transplantation (allo‐HSCT). These models primarily focus on pre‐transplant factors, while algorithms incorporating changes in patient's status post‐allo‐HSCT are lacking. The aim of this study was to develop a predictive soft computing model assessing survival outcomes in allo‐HSCT recipients. In this study, we assembled a comprehensive database comprising of 564 consecutive adult patients who underwent allo‐HSCT between 2015 and 2024. Our algorithm selectively considers critical parameters from the database, ranking and evaluating them based on their impact on patient outcomes. By utilising the Data Ensemble Refinement Greedy Algorithm, we developed an AI model with 93.26% accuracy in predicting survivorship status in allo‐HSCT recipients. Our model used only seven parameters, including age, disease, disease phase, creatinine levels at day 2 post‐allo‐HSCT, platelet engraftment, acute graft‐versus‐host disease (GvHD) and chronic GvHD. External validation of our AI model is considered essential. Machine learning algorithms have the potential to improve the prediction of long‐term survival outcomes for patients undergoing allo‐HSCT.

## 1 Introduction

Allogeneic haematopoietic stem cell transplantation (allo‐HSCT) constitutes a potentially curative treatment option for a range of haematological disorders, both malignant and benign. Nevertheless, the efficacy of allo‐HSCT is often disrupted by numerous complications including, endothelial injury syndromes, such as HSCT‐associated thrombotic microangiopathy, graft‐versus‐host disease (GvHD), relapse of malignancy and occurrence of infections, all of which contribute to lower transplant‐related survival rates [1]. Specifically, acute (aGvHD) and chronic (cGvHD) have been recognised as major causes of death in allo‐HSCT recipients, followed by cardiovascular disease [2].

Continuous development of several laboratory or clinical biomarkers and their integration into routine clinical practice has led to the identification of patients at elevated risk for organ dysfunction‐related complications and those likely to experience reduced overall survival (OS) [3]. Moreover, various scoring systems have significantly contributed to this objective, thus enabling more precise and effective clinical interventions. Notable examples include the Disease Risk Index (DRI) for allo‐HSCT, which categorises patients into four groups based on diagnosis, disease stage and cytogenetic abnormalities to predict their two‐year OS and the Haematopoietic Cell Transplantation‐specific Comorbidity Index (HCT‐CI) that serves as an evaluative tool for predicting survival following HSCT in individuals with hematologic malignancies [4, 5]. Additionally, the Endothelial Activation and Stress Index (EASIX) has been demonstrated as a useful prognostic instrument for estimating the development of GvHD and OS in these patients [6]. Despite these advances, the predictive accuracy of these scoring systems remains suboptimal. Consequently, there is a pressing need for the development of novel models to more accurately predict outcomes and OS in allo‐HSCT recipients.

In recent years, machine learning (ML) techniques have been extensively applied in the field of HSCT [7]. Artificial intelligence (AI) models have been utilised to predict allo‐HSCT‐related complications, including thrombotic events, bloodstream infections, kidney injury, GvHD and pulmonary complications [7]. Moreover, these advanced approaches have facilitated the prediction of haematopoietic stem cell mobilisation in allogeneic donors, ensuring optimal timing for HSCT in patients with haematological malignancies [8]. ML‐based scoring systems have also proven useful in selecting the most appropriate conditioning regimens for HSCT recipients [9]. Similar predictive models have been developed for overall and leukaemia‐free survival in patients with myelodysplastic syndromes, by utilising clinical and laboratory variables [10].

While current models predominantly use baseline, pre‐transplant characteristics, there is a lack of algorithms that account for variables post‐HSCT. Moreover, the accuracy of most of these algorithms remains suboptimal in the prediction of mortality after transplantation. To address this gap, we aimed to develop an algorithm for predicting survival outcomes in our real‐world cohort of allo‐HSCT recipients by incorporating both baseline clinical characteristics and post‐transplantation changes.

## 2 Materials and Methods

### 2.1 Study Design and Population

The primary aim of our work was to construct an AI model for the prediction of long‐term survival post‐allo‐HSCT. As mentioned above we aimed to include in our model both pre‐ and post‐allo‐HSCT variables and transplantation‐associated characteristics. Thus, we conducted this retrospective observational study. A database comprising 564 consecutive adult patients, who underwent allo‐HSCT in our JACIE (Joint Accreditation Committee‐ISCT & EBMT) accredited center in the Haematology department of George Papanikolaou Hospital between 2015 and 2024, was compiled to predict survivorship rates. The database is appended to this paper as Appendix S1 in the Excel file entitled ‘*Database*’.

Patient gender, age, haematological disease, disease phase, donor type, HLA matching with the donor, graft source, conditioning regimen toxicity, number of CD34+ cells infused, laboratory markers post‐allo‐HSCT (platelets/lactate dehydrogenase/creatinine at Day 2), neutrophil/platelet engraftment and the development of aGVHD/cGVHD and secondary malignancy during follow‐up were retrieved. Allo‐HSCT was performed based on the standard operating procedures of the European Society for Blood and Marrow Transplantation (EBMT), while all patients were meticulously evaluated before their admission to the unit [11]. The conditioning regimen was considered myeloablative in patients who were treated with any of the following: total body irradiation > 8 Gy, melphalan > 140 mg/m^2^, oral busulfan ≥ 9 mg/kg, intravenous busulfan ≥ 7.2 mg/kg or thiotepa ≥ 10 mg/kg [11]. In the other cases, it was considered a reduced‐intensity regimen. DRI was retrospectively assessed based on the haematological disease, disease phase, and cytogenetic abnormalities identified before HSCT [4]. Patients with aplastic anaemia were considered an intermediate DRI subcategory. Assessment and grading of acute GVHD were performed according to the criteria of Glucksberg et al. [11], while chronic GVHD was assessed and graded according to the 2014 National Health Institute criteria. Additionally, survival status during follow‐up was evaluated (Alive, Dead, Alive but follow‐up less than 24 months). Our study protocol has been approved by the Institutional Review Boards of the George Papanicolaou Hospital and conducted in accordance with the Declaration of Helsinki.

**FIGURE 1:**
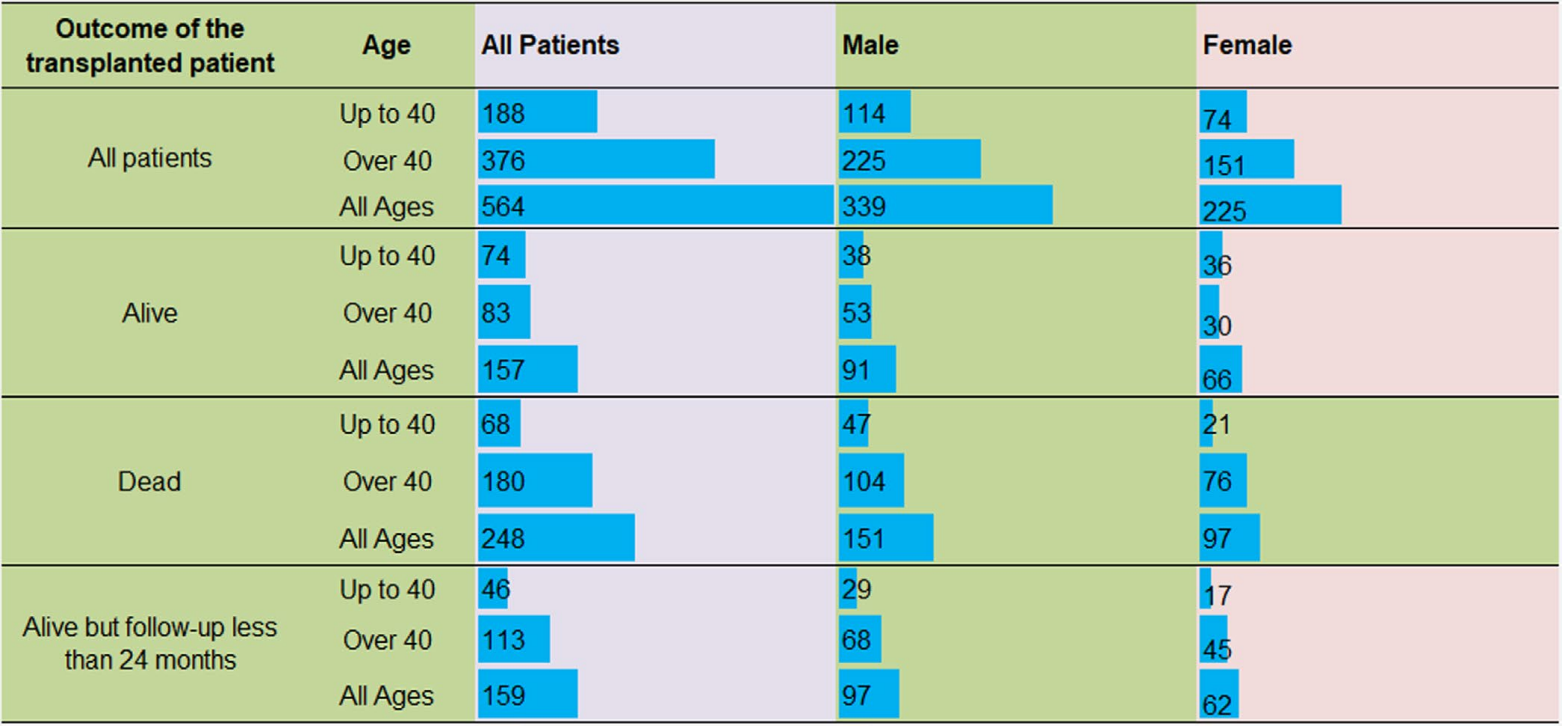
Number of transplanted patients categorised by age, gender and outcome of the survivorship in adult allogeneic haematopoietic cell recipients.

The descriptive statistics of the population are presented in Table 1. Furthermore, in Figure 1 a detailed analysis of the studied population is provided. Data is presented for the entire cohort of transplanted patients and for three subcategories based on the outcome (Alive, Dead and Alive but follow‐up less than 24 months) as well as by age groups (up to 40 and over 40). Moreover, these categorisations are presented independently of gender and by gender (Male or Female). These classifications based on the outcome, gender and age result in a total of 12 patients' categories. Despite the considerable number of these 12 categories, it is crucial to note that each category contains a satisfactory number of individuals, thus ensuring a robust dataset. Notably, no category has fewer than 17 transplanted patients, thus underscoring the reliability of this database.

**TABLE 1:** Descriptive statistics of allo‐HSCT recipients.

Patients' characteristics	
**Gender, *N* (%)**	
Male	339 (60.1)
Female	225 (39.9)
**Age, median**	47 (18–88)
**Disease, *N* (%)**	
(1) ALL, HL, NHL, Prolymphocytic leukaemia, MPAL	153 (27.1)
(2) AML, MDS, MDS/MPN, MF	367 (65.1)
(3) Aplastic anaemia, autoimmune, CLL, CML, MM, plasma cell disorder, other	44 (7.8)
**Disease phase, *N* (%)**	
(1) CR1, CR2, CR3, CR1 MRD+, PR, VGPR, Prim. Ref. Chemosensitive, Relapse Chemosensitive	447 (79.3)
(2) Prim. ref. chemoresistant, relapse chemoresistant, active disease, refractory, severe, very severe, refractory progressive, graft failure	117 (20.7)
**Donor type, *N* (%)**	
Sibling	204 (36.2)
Unrelated	284 (50.4)
Haploidentical	76 (13.5)
**HLA matching**	
(1) 8/8, 10/10, 12/12, 9/10,6/6	458 (81.2)
(1) 7/8,5/6, Haploidentical	106 (18.8)
**Graft source**	
Peripheral	514 (91.1)
Bone marrow	47 (8.3)
Other source	3 (0.5)
**Conditioning regimen toxicity**	
Myeloablative	218 (38.7)
Reduced intensity	346 (61.3)
**Platelets at Day 2 post‐allo‐HSCT, median**	61.6 × 10^9^ (3 × 10^9^ − 922.2 × 10^9^)
**Lactate dehydrogenase (mg/dl) at Day 2 post‐allo‐HSCT, median**	180 (14–4326)
**Creatinine (mg/dl) at Day 2 post‐allo‐HSCT, median (range)**	2.6 (0.6–5.3)
**CD34 + x12^6^/Kg cells infused, median**	6 (1.23–19.8)
**Neutrophil engraftment, *N* (%)**	558 (98.9)
**Platelet engraftment, *N* (%)**	512 (90.8)
**DRI**	
Low	260 (46.1)
Intermediate	167 (29.6)
High	137 (24.3)
**Acute GvHD, *N* (%)**	
Grade 0 or I	219 (38.8)
Other grade	345 (61.2)
**Chronic GvHD, *N* (%)**	276 (48.9)
**Secondary malignancy, *N* (%)**	10 (1.8)

Abbreviations: ALL, acute lymphoblastic leukaemia; Allo‐HSCT, allogeneic haematopoietic stem cell transplantation; AML, acute myeloid leukaemia; CLL, chronic leukocytic leukaemia; CML, chronic myelogenous leukaemia; CR, complete remission; DRI, Disease Risk Index; GvHD, graft versus host disease; HL, Hodgkin lymphoma; HLA, human leukocyte antigen; MDS, myelodysplastic syndrome; MF, myelofibrosis; MM, multiple myeloma; MPAL, mixed‐phenotype acute leukaemia; MPN, myeloproliferative neoplasm; MRD, minimal residual disease; NHL, non‐Hodgkin lymphoma; PR, partial remission; PRIM. REF., primary refractory; VGPR, very good partial response.

### 2.2 AI Model to Reveal the Parameters Affecting the Outcome of the Allo‐HSCT Recipients

The main aim of this study, as mentioned above, was to develop a predictive soft computing model capable of reliably assessing survival outcomes in allo‐HSCT recipients. It is important to underline that this objective entails the design of an algorithm that selectively considers only the critical from the pool of 18 total parameters in the database, as presented in Table 1, which determines the survivorship in allo‐HSCT. The variables selected, both clinical and laboratory, are widely available and evaluated in most allo‐HCT recipients. Thus, this model would be easily applied to an allo‐HCT setting to predict survival in these patients. Moreover, it is of particular importance for the algorithm to evaluate and rank these parameters based on their impact on the outcome of the transplanted individuals.

This task is particularly challenging due to the large number of possible parameter combinations that need to be examined during the model design, ranging from patterns involving 1 parameter to those involving up to 18. The number of possible combinations is given by the following equation:

(1) $$\text{Patterns Combinations}=2\sum_{i=1}^{n}\frac{n!}{i!\left(n-i\right)!}=2\left(2^{n}-1\right)$$

where *n* is the number of database parameters.

To address this challenge, DERGA algorithm (Data Ensemble Refinement Greedy Algorithm) was employed, a methodology introduced and successfully applied by our group in the prognosis of COVID‐19 severity by utilising haematological markers and elucidating the genetic background of COVID‐19 patient outcomes [12, 13].

Briefly, the fundamental principle of this algorithm involves initially constructing and training a model incorporating all 18 parameters. Subsequently, 18 distinct models are developed and trained, each excluding 1 of the 18 parameters. The model exhibiting the least accurate predictions indicates that the excluded parameter exerts the least influence among the 18 parameters in assessing the survival outcomes. This process is then repeated with the remaining 17 parameters. Consequently, the sequential elimination of 1 parameter at a time results in models exhibiting progressively enhanced accuracy until reaching a threshold, beyond which accuracy diminishes. The step at which maximal accuracy is attained delineates the optimal pattern predicting the survival outcomes in allo‐HSCT recipients.

The advantages of this algorithm encompass achieving optimal prediction accuracy, identifying the minimal set of parameters along with their corresponding pattern that defines the prediction and ranking the respective parameters based on their importance in predicting survival outcomes among allo‐HSCT recipients. DERGA also offers reduced complexity, as the total combinations of parameter patterns necessitating examination are determined by the following equation:

(2) $$\text{Patterns Combinations}=\frac{n\left(n+1\right)}{2}$$

A significantly lower figure compared to the potential combinations outlined in Equation (1).

For each pattern of input parameters derived from the DERGA algorithm, corresponding predictive models were designed and trained utilising a set of classification meta‐algorithms accessible within the literature. The comprehensive elucidation of this process will be presented in the results section.

The proposed DERGA algorithm outperforms other algorithms because it incorporates all of them. Specifically, the first step of the proposed DERGA algorithm, during which all features (input parameters) are included (i.e., the process of removing non‐crucial input parameters has not yet begun), corresponds to the results that would be obtained for each individual algorithm. Consequently, one of the main advantages of the proposed algorithm is that it can encompass all available classification algorithms and lead to better outcomes.

## 3 Results

The optimal combination of parameters determining the outcome of the transplanted individuals (Alive, Dead and Alive but follow‐up less than 24 months) was investigated. Specifically, using the database of 564 allo‐HSCT recipients, the proposed DERGA algorithm was implemented with five established classification meta‐algorithms available in the literature. These meta‐algorithms included Extra Trees, Decision Trees, Cat Boost, Gradient Boosting and Adaptive Boosting (AdaBoost) [14, 15, 16, 17, 18].

The computational predictive models were trained by utilising the 564‐patient database, with each dataset consisting of 18 parameters (Table 1). The database was partitioned into two subsets: one for training the forecasting models (training datasets) consisting of 451 datasets (80% of the total database) and one for testing the reliability of these models, consisting of the remaining 113 datasets (20% of the total database). These two datasets corresponded to two cohorts, one for developing the computational predictive model and the other for testing the model's reliability and result confirmation.

Through the proposed algorithm, a total of 2,158,875 models were developed and trained, corresponding to 431,775 models for each of the five classification meta‐algorithms used. The number of 431,775 models was calculated as follows: 171 patterns (as defined by Equation 2 for 18 input parameters) × 25 random splits of the dataset into training and testing sets × 101 random seeds used for each optimisation algorithm. All predictive models were assessed using classical and widely accepted performance indices such as Accuracy, Precision, F1‐Score and Recall [19]. The best models for each of the five classification meta‐algorithms are presented in the Appendix S1 Excel file ‘*Best Models*’.

**FIGURE 2:**
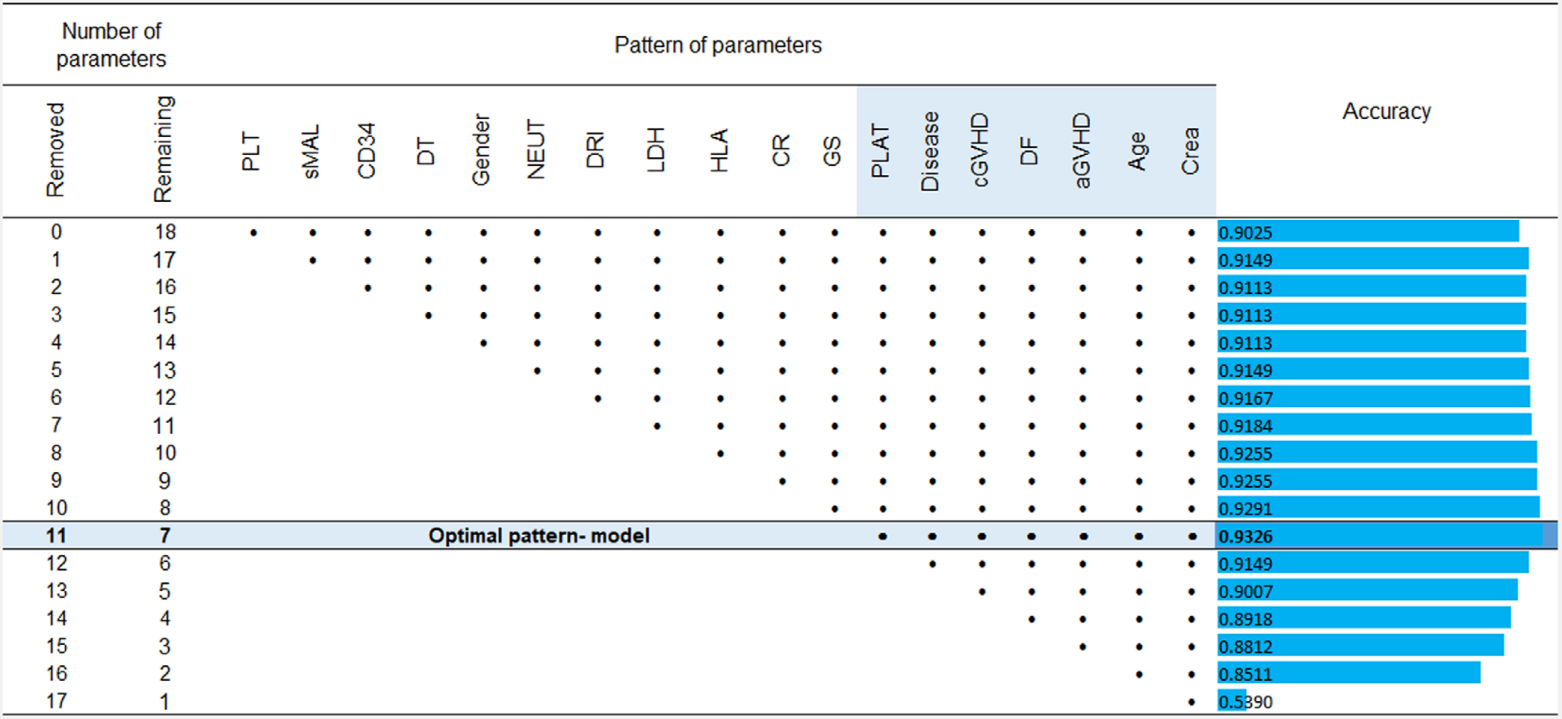
Accuracy of optimal DERGA‐Extra Trees model (Bullet symbol (•) in the column of a parameter means that this parameter participates as input parameter in the forecasting computational model). aGVHD, acute Graft‐versus‐host disease; CD34, CD34 cells infused; cGVHD, graft‐versus‐host disease; CR, conditioning regimen; Crea, creatinine at day 2 post‐allo‐HSCT; DF, disease phase; DRI, disease risk index; DT, donor type; GS, graft source; HLA, HLA matching; LDH, lactate dehydrogenase at day 2 post‐allo‐HSCT; NEUT, neutrophil engraftment; PLAT, platelet engraftment; PLT, platelets at day 2 post‐allo‐HSCT; sMAL, secondary malignancy.

In Figure 2, the results for the optimal mathematical model designed and trained using the DERGA algorithm are presented, corresponding to the Extra Trees classification meta‐algorithm. This optimal prediction model achieved an accuracy of 0.9326, utilising only 7 of the 18 total parameters in the database. The same figure, along with all performance indices used, is provided as Appendix S1 in the Excel file entitled ‘*Optimal DERGA‐Extra Trees Model*’.

The seven parameters and their influence on the survival of a transplanted individual are the following:

1. Creatinine at Day 2 post‐allo‐HSCT
2. Age
3. aGVHD
4. Disease phase
5. cGVHD
6. Disease
7. Platelet engraftment

## 4 Discussion

In the present study, we developed an AI model achieving a 93.26% accuracy rate in predicting long‐term survival in a real‐world cohort of adult allo‐HSCT recipients. This model incorporates the seven most influential clinical variables, after ranking them, including creatinine at Day 2, patients' age, aGVHD, disease phase, cGVHD, haematological disease and platelet engraftment. To the best of our knowledge, this is the first AI model to encompass both pre‐ (age, disease and disease phase) and post‐HSCT variables for predicting mortality in adult HSCT recipients with such a high accuracy. Furthermore, the accuracy of our AI model in the prediction of long‐term survival is one of the highest reported in the literature. In previous works of our group, the factors incorporated in this model have been recognised as predictors of OS [20, 21].

In our AI model, a 93.26% accuracy was exhibited in the prediction of survival status in allo‐HSCT patients. Moreover, survival in our model was not treated as a time dependent event. Random survival forests were identified through a systematic review, in which 24 studies were included, as the top‐performing ML algorithm for survival prediction, with an area under the curve (AUC) of 0.72 as shown in receiver operating characteristic (ROC) analysis [22]. Shouval et al. [23], in their study, constructed an ML model (alternating Decision Tree algorithm) for predicting transplant‐associated mortality in patients with acute leukaemia by using 10 pre‐transplant variables and achieving an AUC of 0.702 at 100 days after the HSCT. In this study 28,236 adult HSCT recipients were included. Moreover, the same group of researchers created another ML algorithm with an in silico approach, predicting non‐relapse mortality 100 days post‐HSCT (AUC = 0.67) [24]. The most important variables predicting outcomes in the algorithm were disease stage, donor type and conditioning regimen. Eisenberg's et al. [25] Gradient Boosting Machine (GBM) model managed to predict 21‐day mortality, with an AUC of 0.92, and cytomegalovirus reactivation, with an AUC of 0.83, by encompassing time‐dependent clinical and laboratory data. Unlike these studies, our methodology focused on the likelihood of long‐term survival rates, with our model achieving a significantly higher accuracy. However, our single‐center study, had a smaller number of patients included, in comparison to those described above. Thus, external validation of our algorithm is considered essential.

Interestingly, Okamura et al. [26] developed an interactive web application using ML to predict 1‐year OS in allo‐HSCT recipients, achieving an AUC of 0.70. Chois et al.'s [27] GBM algorithm predicted 5‐year survival post‐HSCT with an AUC of 0.788, based on pre‐transplant clinical and laboratory characteristics of donors and patients. Mussetti et al. [28] aimed to predict 2‐year mortality in a large cohort of patients with an AUC of 0.64. These approaches mainly focused on pre‐ and transplant‐associated variables in the prediction of long‐term survival after the transplantation procedure, while in our algorithm post‐allo‐HSCT factors were included.

Zhou and colleagues were the first to construct an ML model for the prediction of survivorship status long term after the transplantation, incorporating both pre‐nd post‐allo‐HSCT variables, while in their study only children and young adults were included [29]. In Table 2, the findings of the studies examining the role of AI predictive models in survival outcomes of adult allo‐HCT patients are summarised. Echecopar et al. [30] constructed another ML algorithm predicting 1‐year survival in paediatric patients with 72% accuracy, incorporating variables like diagnosis and donor type. In our study, exclusively adult patients were included, and the efficacy of our AI model in the survival prediction of children and adolescents who undergo allo‐HSCT should be examined in future research.

**TABLE 2:** Artificial intelligence studies in the prediction of survival outcomes in allo‐HCT recipients.

First author, year of publication [References]	Number of patients	Variables selected	Outcome	AUC of the model
Shouval, 2015 [23]	28,236 patients	Disease stage, Karnofsky at HCT, Age, days between diagnosis and HCT, conditioning regimen, donor type, annual HCT experience, year of HCT, donor‐recipient CMV serostatus combination, diagnosis	Prediction of survival 100 days post‐allo‐HCT	0.702
Shouval, 2016 [24]	25,923 acute leukaemia patients who received allo‐HCT	The top 3 ranking variables, in all proposed algorithms were disease stage, donor type, and conditioning regimen	Prediction of non‐relapse mortality 100 days post‐allo‐HCT	0.670
Eisenberg, 2022 [25]	2191 allo‐HCT recipients	The most significant variables were the day of the prediction (days after HCT), C‐reactive protein, blood urea nitrogen, glutamate oxaloacetatetransaminase, and protein levels	Prediction of mortality 21 days post‐allo‐HCT	0.92 for prediction of 21 days post‐HCT survival
Okamura, 2021 [26]	363 allo‐HCT recipients	Age, conditioning regimen intensity, DRI, HLA 8 allele compatibility, donor source, performance status, HCT‐CI, number of transplantations	Prediction of 1‐year overall survival, progression‐free survival, relapse/progression, and nonrelapse mortality	The AUCs for 1‐year overall survival, progression‐free survival, relapse/progression, and nonrelapse mortality were 0.70, 0.72, 0.73, and 0.77, respectively
Choi, 2022 [27]	1470 allo‐HCT recipients	Diagnosis and disease, disease risk, WBC count at diagnosis, extramedullary disease at diagnosis, extramedullary disease at HCT, karyotype at diagnosis, karyotype at HCT, CMV serostatus of recipient and donor, hepatic score of HCT‐CI, HCT‐CI total score, conditioning regimen, donor type, recipient HLA type, donor HLA type, RBC transfusion before HCT, platelet transfusion before HCT	Prediction of 5‐year survival post‐allo‐HCT	0.788
Mussetti, 2024 [28]	33,927 allo‐HCT patients	Clinical and laboratory variables	Prediction of 2‐year overall mortality	0.64
Zhou, 2024 [29]	St Jude cohort (*N* = 738) and the MSKCC cohort (*N* = 218)	Various pre‐ and post‐allo‐HTC variables were included	Prediction of 1‐ and 2‐year survival	0.745 (for 1‐year survival) 0.722 (for 2 years survival)

Abbreviations: allo‐HCT, allogeneic haematopoietic cell transplantation; AUC, area under the curve; CMV, cytomegalovirus; DRI, disease risk index; haematopoietic cell transplantation‐specific comorbidity index; HCT, haematopoietic cell transplantation; RBC, red blood cells; WBC, white blood cells.

Various ML approaches have predicted HSCT outcomes such as malignancy relapse. Arabyarmohammadi et al. [31] created an ML model to predict relapse post‐HSCT using cytologic aspirate image markers from AML patients (AUC in the ROC analysis 0.71 in the validation cohort). Afanaseva et al. [32] in their pilot study developed an ML algorithm for the prediction of relapse post‐allo‐HSCT in adult patients with Ph‐positive ALL, based on post‐transplant characteristics. A GBM method, using the highest BCR/ABL1 levels, as measured in different time points, presence of GvHD, time of prediction (days after HSCT), BCR/ABL1 levels at the time of prediction, and administration or not of tyrosine kinase inhibitors, was developed with a sensitivity of 0.91 to predict relapse in this population. Assessing the performance of these algorithms in real‐world clinical settings has the potential to significantly enhance their practical utility and effectiveness.

Our model incorporated GvHD (both acute and chronic) occurrence as a predictor of long‐term survivorship. Previous ML algorithms have predicted GvHD and thrombotic complications post‐HSCT. Salehnasab et al. [33] created a GBM algorithm predicting aGvHD with an AUC of 0.91, using routinely used biomarkers like albumin, uric acid and C‐reactive protein. It is considered crucial to examine the accuracy of these models in real‐world clinical settings in order to incorporate them in everyday practice. Furthermore, we plan to examine the efficacy of the DERGA algorithm, based on both molecular and clinical variables, in other transplant‐related outcomes, such as HSCT‐TMA.

Applications of AI have been implemented also in the prediction of outcomes in patients who receive chimeric antigen receptor‐T (CAR‐T) cell immunotherapy, and especially in early identification of cytokine release syndrome CRS, a major complication of this treatment approach [34, 35]. Recently, a computational approach, using the R programming language, has been developed for grading CAR‐T‐related toxicities [36].

The study's limitations include its retrospective nature, the single‐center data, and the focus on adult patients with haematological malignancies, necessitating validation in diverse populations and paediatric patients. Despite the high accuracy of the predictive model developed in this study, the authors emphasise the need to update the database with more data to enhance the model's accuracy for better clinical decision‐making. Moreover, the accuracy of our AI model was not compared to a conventional Cox regression analysis model for survival.

Conclusively, this study demonstrates the effectiveness of DERGA algorithm in achieving high prediction accuracy (93.26%) regarding long‐term survival outcomes in adult al‐lo‐HSCT recipients. The use of well‐established classification algorithms from the ML literature, orchestrated in a data ensemble refinement procedure, enables the identification of the minimal set of parameters and their corresponding patterns that define the optimal prediction model. Future research should prioritise the external validation of our AI predictive model by independent HSCT centers to ensure its robustness and applicability across diverse patient populations. Additionally, further efforts should be made to expand and update the current database to encompass a larger and more comprehensive dataset. This would ensure adequate representation of all possible parameter values relevant to the studied domain, thereby enhancing the model's generalisability and reliability. Moreover, subsequent studies should aim to extend the predictive scope of AI models to include other HSCT‐related complications, which remain a critical area of clinical importance. AI algorithms, such as ours, also can be helpful for the development of novel molecular therapeutics [37].

In response to the potential limitations highlighted, this study acknowledges the retrospective nature of the dataset, which may introduce biases such as confounding factors and data imbalance. These limitations underline the importance of designing future prospective studies that proactively address these issues. Prospective studies should aim to curate balanced and representative datasets, implement robust techniques to mitigate selection bias, and ensure the inclusion of diverse patient populations to enhance the predictive accuracy and fairness of the model. Furthermore, this study recognises the need to integrate the DERGA algorithm into clinical workflows effectively. Future research should explore strategies to overcome potential barriers, such as clinician training, system interoperability and real‐time application challenges, to facilitate its adoption in routine clinical practice. These considerations will not only improve the model's usability but also maximise its impact in improving patient outcomes.

## Author Contributions

**Panagiotis G. Asteris:** conceptualization (equal), methodology (equal), software (equal), supervision (equal), visualization (equal), writing – review and editing (equal). **Danial J. Armaghani:** methodology (equal). **Amir H. Gandomi:** methodology (equal). **Ahmed Salih Mohammed:** methodology (equal). **Zoi Bousiou:** methodology (equal). **Ioannis Batsis:** methodology (equal). **Nikolaos Spyridis:** methodology (equal). **Georgios Karavalakis:** methodology (equal). **Anna Vardi:** methodology (equal). **Markos Z. Tsoukals:** methodology (equal), software (equal). **Leonidas Triantafyllidis:** methodology (equal), software (equal). **Evangelos I. Koutras:** methodology (equal), software (equal). **Nikos Zygouris:** methodology (equal). **Georgios A. Drosopoulos:** methodology (equal). **Leonidas Dritsas:** methodology (equal). **Nikolaos A. Fountas:** methodology (equal). **Nikolaos M. Vaxevanidis:** methodology (equal). **Abidhan Bardhan:** methodology (equal). **Pijush Samui:** methodology (equal). **George D. Hatzigeorgiou:** methodology (equal). **Jian Zhou:** methodology (equal). **Konstantina V. Leontari:** methodology (equal). **Paschalis Evangelidis:** methodology (equal). **Nikolaos Kotsiou:** methodology (equal), writing – original draft (equal). **Ioanna Sakellari:** methodology (equal), supervision (equal), writing – review and editing (equal). **Eleni Gavriilaki:** conceptualization (equal), formal analysis (equal), project administration (equal), writing – review and editing (equal).

## Conflicts of Interest

The authors declare no conflicts of interest.

## Supporting information


Appendix S1.

## Data Availability

The authors declare that the data supporting the findings of this study are available within the paper.
